# The Battle Against Biofilms: A Focus on Novel Antimicrobial Strategies and Their Mechanisms of Action

**DOI:** 10.3390/antibiotics14020111

**Published:** 2025-01-21

**Authors:** Efstathios Giaouris, Olivier Habimana

**Affiliations:** 1 Department of Food Science and Nutrition, School of the Environment, University of the Aegean, 81400 Myrina, Lemnos, Greece; 2 Department of Biotechnology and Food Engineering, Guangdong Technion—Israel Institute of Technology, Shantou 515063, China; 3 Faculty of Biotechnology and Food Engineering, Technion—Israel Institute of Technology, Haifa 3200003, Israel

Biofilms represent the predominant mode of microbial growth across a variety of environments, encompassing both natural and anthropogenic settings [1]. These protect the enclosed cells from environmental perturbations, including physicochemical stresses, antibiotics, and other biocide exposures [2,3]. In addition to their beneficial roles, mainly with respect to their crucial involvement in environmental sustainability issues (e.g., bioremediation, wastewater treatment, bioreactors producing beneficial compounds, other chemicals, and fuels) [4], biofilms are mostly known for the important problems these cause in many areas, including persistent human infections [5], the biofouling of medical devices (e.g., indwelling catheters and prosthetic heart valves) [6], food contamination [7], surface corrosion [8], crop losses [9], problems in marine traffic [10], productivity losses, and considerable increases in energy consumption [11]. The increased recalcitrance of biofilms to current antimicrobials has prompted the search for novel, cost-efficient, and preferable eco-friendly antimicrobial strategies to combat them [12,13]. These strategies should effectively prevent biofilm formation and/or eliminate biofilm cells while minimizing the risk of their developing resistance. The compilation below highlights findings from five recent studies, each providing distinct insights into innovative green strategies for fighting biofilms and resistant microorganisms.

*Staphylococcus epidermidis* is a common nosocomial pathogen usually associated with infections linked to medical devices. This bacterium often forms biofilms that confer resistance to antibiotics and immune responses [14]. The prevalence of multidrug-resistant *S. epidermidis* strains has created a need for novel approaches to antibiotics therapy. The first study by Santativongchai et al. [Contribution 1] examined the potential of octyl gallate (OG), a food-grade antioxidant previously shown to enhance antibiotic activity against *Staphylococcus aureus* [15], in improving the antimicrobial and antibiofilm activities of penicillin and bacitracin against *S. epidermidis*. Octyl gallate significantly lowered the minimum inhibitory concentration (MIC) and minimum bactericidal concentration (MBC) of penicillin, resulting in an 8-fold reduction, and of bacitracin, leading to a 4-fold reduction. The synergy of the subject with antibiotics was validated through the application of checkerboard titration assays, with fractional inhibitory concentration (FIC) and fractional bactericidal concentration (FBC) indices ≤ 0.5. Additionally, OG, along with low concentrations of each of the two antibiotics, significantly reduced biofilm formation by *S. epidermidis* through the inhibition of microcolony development. Regarding its mode of action, it was found that OG increased bacterial cell wall permeability, improving penicillin and bacitracin’s ability to access their cellular targets. However, OG’s synergistic effects were specific to penicillin and bacitracin and were not observed with other antibiotics, such as ciprofloxacin or tetracycline. The study demonstrated that OG could act as a potent drug potentiator, enhancing the antimicrobial and antibiofilm activities of penicillin and bacitracin against *S. epidermidis*. This suggests that OG might be utilized in the development of novel adjuvant strategies to combat antibiotic-resistant *S. epidermidis* infections, particularly in biofilm-associated conditions.

*Salmonella enterica* constitutes a significant foodborne pathogen implicated in gastroenteritis and systemic infections. Its ability to form biofilms on biotic and abiotic surfaces enhances its resistance to antibiotics and disinfectants [16]. The biofilms produced by *Salmonella* are characterized by a high concentration of extracellular polymeric substances (EPS), predominantly composed of amyloid curli and cellulose, both of which play a significant role in their persistence. Raspberry Ketone (RK) is a natural flavoring agent that has demonstrated potential bioactive properties, including the mitigation of obesity-induced Alzheimer’s disease [17]. The second study, conducted by Farha et al. [Contribution 2], examined the potential antibiofilm properties of RK against *S. enterica* Typhimurium. The administration of RK at a concentration of 200 µg/mL effectively inhibited biofilm formation by *S*. Typhimurium while not significantly impacting the proliferation of planktonic cells. This intervention disrupted the *rdar* (red, dry, and rough) morphotype, which is associated with curli fimbriae and cellulose, in *Salmonella* on a Congo red agar plate, reduced pellicle formation at the air–liquid interface, and decreased the density of multicellular aggregates. Although RK diminished cellulose levels in biofilms, as evidenced by the decreased fluorescence of Calcofluor-stained samples, no significant changes were observed in the expression of genes directly related to cellulose biosynthesis. Furthermore, both swimming and swarming motility were significantly impaired in *S*. Typhimurium treated with RK, which likely contributed to the observed reduction in biofilm formation. Quantitative proteomics revealed 128 differentially expressed proteins in biofilms treated with RK. The pathways impacted included the production of amyloid curli, flagellar assembly, bacterial invasion mechanisms, and arginine biosynthesis. Transcriptional analysis validated the downregulation of the key genes associated with biofilm development, specifically *csgD* and *csgB*. Collectively, these findings establish RK as a promising food-safe antimicrobial agent for the mitigation of *Salmonella* biofilm formation in clinical and industrial settings.

The coexistence of bacterial and fungal pathogens, including *S. aureus* and *Candida albicans*, within mixed biofilms significantly enhances resistance to antimicrobial treatments and may contribute to the persistence of chronic infections [18]. Metal nanoparticles (NPs), particularly those composed of gold and silver, have been the subject of investigation due to their antimicrobial properties, attributed to their capacity to disrupt microbial cell membranes and biofilm matrices. β-caryophyllene, a plant-derived volatile sesquiterpene, is utilized as a flavoring agent in the food industry and is recognized for its biocompatibility and antimicrobial activities. The research conducted by Khan et al. [Contribution 3] aimed to evaluate the antimicrobial and antibiofilm properties of gold nanoparticles (AuNPs) synthesized using β-caryophyllene as a reducing agent (β-c-AuNPs) against mixed biofilms of *S. aureus* and *C. albicans*. The study focused on testing the efficacy of β-c-AuNPs in both inhibiting biofilm formation and eradicating mature biofilms. The synthesized β-c-AuNPs exhibited an average size of 17.6 ± 1.2 nm, an irregular shape, and a high negative zeta potential, indicating stability. The MIC of the β-c-AuNPs against both pathogens was determined to be 512 µg/mL, which is significantly lower than that obtained for β-caryophyllene alone, thus demonstrating enhanced antimicrobial activity. β-c-AuNPs effectively inhibited the initial biofilm formation of *S. aureus*, *C. albicans*, and their mixed cultures in a concentration-dependent manner. However, their inhibitory effect on mixed biofilms was found to be less pronounced compared to that on single-species biofilms. Moreover, β-c-AuNPs significantly diminished the colony-forming units (CFU) of mature biofilms at the MIC and at higher concentrations. An analysis using scanning electron microscopy (SEM) revealed reduced surface adherence and biofilm density for both single-species and mixed-species biofilms following treatment with β-c-AuNPs. Consequently, these green NPs exhibit considerable potential as a novel therapeutic strategy for addressing biofilm-associated polymicrobial infections.

Eugenol is a naturally occurring phenolic compound present in cinnamon, clove and various other botanical sources, known for its antimicrobial, antioxidant, and other functional properties [19]. It demonstrates notable antimicrobial efficacy, with an MIC ranging from 0.0312 to 8 µg/mL and an MBC ranging from 0.0625 to 16 µg/mL, against a plethora of pathogens. This antimicrobial activity is primarily attributable to its ability to alter cell membrane permeability, which results in intracellular content leakage and subsequent microbial death. Nonetheless, the high volatility and low water-solubility of eugenol significantly constrain its practical effectiveness. Furthermore, prolonged exposure or elevated dosages may lead to toxicity and allergenic responses. In response to these critical limitations, approaches such as loading eugenol into polymers or polymerizing eugenol-derived monomers have been developed. These strategies not only enhance stability and reduce volatility and cytotoxicity but also prolong the antimicrobial action of eugenol. Such modifications can also yield value-added materials, including materials with self-healing properties, improved thermal stability, and increased water-resistance, thereby rendering them suitable for diverse promising applications of eugenol. The review study conducted by Kowalewska and Majewska-Smolarek [Contribution 4] highlights the versatility of eugenol-based polymeric materials in controlling bacterial biofilms and improving material properties. Indeed, these materials hold significant potential for applications across healthcare, food packaging, and sustainable environmental technologies. Interestingly, eugenol-derived polymeric films were shown to effectively prevent and disrupt biofilms, particularly on stainless-steel surfaces. Moreover, polymeric films containing eugenol were shown to significantly reduce bacterial adhesion and inhibit biofilm formation on food packaging materials, thereby extending the shelf-life of food products. In comparison to free eugenol, these eugenol-based polymeric materials also displayed reduced cytotoxicity, rendering them suitable for biomedical applications such as wound dressings and coatings for medical devices.

*Campylobacter* spp. are recognized as prominent causes of foodborne gastroenteritis worldwide, primarily associated with the consumption of raw or undercooked poultry. Their ability to form biofilms on abiotic surfaces increases their extraintestinal survival and resistance to antimicrobials [20]. Alarmingly, the prevalence of antimicrobial resistance among *Campylobacter* spp. poses a significant challenge to the effectiveness of conventional treatments, thereby underscoring the necessity of alternative approaches. The final study by Kostoglou et al. [Contribution 5] examined the antibacterial and antibiofilm properties of benzalkonium chloride (BAC), erythromycin (ERY), and *L*(*+*)-lactic acid (LA) against 12 *Campylobacter* spp. isolates obtained from raw chicken meat. The MIC and MBC values for BAC ranged from 0.5 to 32 µg/mL depending on the medium. Conversely, ERY’s MIC and MBC values exhibited a considerable range (0.25–1024 µg/mL), with a high level of resistance observed in certain isolates. Lactic acid showed MICs and MBCs ranging from 1024 to 2048 µg/mL, with increased resistance noted in the presence of blood. Benzalkonium chloride demonstrated efficacy in inhibiting biofilm formation in monoculture conditions, with minimum biofilm inhibitory concentration (MBIC) values that were equal to or surpassed the MICs for planktonic cells. Erythromycin also proved effective against biofilms, including those formed by resistant isolates; however, a significantly higher dose (32 μg/mL) was required. Regarding LA, a naturally occurring organic acid found in fermented foods such as yogurt, sauerkraut, and pickles, its MBICs against monocultures varied between 1024 and 2048 µg/mL. However, mixed biofilms, consisting of three different *Campylobacter* isolates that were co-cultured, exhibited heightened tolerance, requiring LA concentrations of 4096 µg/mL for biofilm inhibition, thereby emphasizing the protective effect of microbial interactions. The study accentuates the importance of developing customized antimicrobial strategies, as the individual effectiveness of the three antimicrobial agents tested varied significantly based on isolate type, growth mode (planktonic or biofilm), intercellular interactions (monocultures or mixed cultures), and the growth medium employed, particularly regarding the presence of blood.

***Critical Analysis of Methodologies and Results: A Synthesis of Antibiofilm Strategies***

This Special Issue features five studies that, despite using various methodologies, collectively highlight key themes related to effective biofilm strategies. A comparative analysis outlines the advantages and limitations of each approach, enhancing our comprehension of the mechanisms of antibiofilm tactics.

***Natural Compounds***: The incorporation of octyl gallate (OG) and raspberry ketone (RK) demonstrates the potential use of natural compounds as antibiofilm agents, particularly when utilized in conjunction with established antibiotics. The mechanism by which OG operates primarily centers on the enhancement of cell wall permeability, thereby synergistically augmenting the efficacy of penicillin and bacitracin in the struggle against *S. epidermidis*. This approach is specific to certain antibiotics, underscoring the need for the careful selection of natural compound–antibiotic pairings [21]. Raspberry ketone, on the other hand, exhibits antibiofilm activity against *S.* Typhimurium by disrupting curli fimbriae and cellulose production, impacting biofilm architecture and motility. The absence of substantial alterations in the expression of cellulose biosynthesis genes indicates a possible indirect mechanism, which may involve post-translational modifications or alternative regulatory processes pathways. While both OG and RK demonstrate promising antibiofilm activity, their efficacy might be limited by their inherent properties, such as OG’s specificity to only certain antibiotics and RK’s potentially lower potency compared to conventional antibiotics.

***Nanomaterials***: β-caryophyllene-derived gold nanoparticles (β-c-AuNPs) demonstrate nanotechnology capabilities in addressing both single and mixed biofilms. Their enhanced antimicrobial activity compared to β-caryophyllene stems from their increased surface area and unique nanoparticle interactions with bacterial membranes and biofilm matrices. Nevertheless, their reduced efficacy against mixed biofilms versus single species underscores the complexities of polymicrobial biofilms and suggests that interspecies interactions may affect antimicrobial effectiveness [22]. The methodology of nanoparticle synthesis and characterization is well-established, but the potential for toxicity and a negative environmental impact requires thorough investigation.

***Polymeric Materials***: The research regarding eugenol-based polymeric materials presents an innovative strategy, mitigating the constraints associated with eugenol’s volatility, cytotoxicity, and its limited solubility in water. The incorporation of eugenol into polymeric matrices enhances its stability and prolongs its antimicrobial action, mitigating its toxicity and facilitating its broader application. This strategy directly tackles the challenge of delivering an effective antimicrobial agent, but its efficacy depends on the choice of polymer and its compatibility with the target application. The reported effectiveness in preventing biofilm formation on stainless-steel surfaces and food packaging materials is encouraging. Nevertheless, a comprehensive testing and characterization of various polymeric formulations is required to enhance their performance and biocompatibility [23].

***Conventional Antimicrobials in Biofilm Contexts***: A study on the antimicrobial effects of benzalkonium chloride (BAC), erythromycin (ERY), and lactic acid (LA) against *Campylobacter* shows varying effectiveness based on isolate type and growth mode. This variance underscores the limitations of conventional antimicrobials in addressing biofilms and highlights the necessity for customized treatment strategies that target specific pathogens [24]. The increased tolerance observed in mixed-species biofilms indicates the protective role of microbial communities. Erythromycin resistance highlights the need for alternative therapies due to the widespread antibiotic resistance observed in *Campylobacter* spp.

***Overall Key Takeaways, Common Themes and Perspectives***

Antimicrobial resistance and biofilm-associated infections undoubtedly represent significant challenges in public health, food safety, and environmental sanitation. In response to these pressing issues, researchers across the globe have been investigating innovative strategies, which include the utilization of natural compounds, nanoparticles, and novel material formulations. Collectively, the studies featured in this Special Issue emphasize the critical necessity for innovative antimicrobial and antibiofilm strategies to address a diverse array of microbial threats. Natural compounds such as octyl gallate (OG), raspberry ketone (RK), and eugenol, alongside nanotechnology-based approaches such as β-c-AuNPs, may present promising avenues for enhancing antimicrobial efficacy and biofilm control. The analysis reveals several important themes in these studies. Firstly, the complexities associated with biofilms necessitate a comprehensive approach, as no singular strategy effectively targets all types of biofilms. Secondly, combining traditional antimicrobials with natural compounds or altering their method of delivery shows great promise for improving their effectiveness. Additionally, utilizing natural compounds, nanoparticles, and polymeric materials opens a viable path for creating novel and eco-friendly antibiofilm approaches. The considerable differences in antimicrobial effectiveness between different microorganisms and growth conditions highlight the need for customized treatment strategies that account for the distinct traits of the biofilm and the pathogen(s) involved. Moreover, the transition from in vitro findings to practical applications necessitates comprehensive in situ and in vivo validation, along with thorough assessments of their safety and scalability, and any issue related to the development of resistance. Future research should therefore prioritize the validation of these findings under real-world conditions to effectively bridge the gap between laboratory studies and practical applications. Investigations into the synergistic effects that may arise from the combination of natural compounds with existing antimicrobials could also yield enhanced solutions to resistant biofilms. Furthermore, exploring the environmental impact of novel antimicrobial materials remains crucial for their sustainable application. Finally, studies on the molecular mechanisms that underpin the efficacy of antibiofilm compounds will not only advance our understanding but also inform the design of more effective antibiofilm strategies. Collectively, these insights will facilitate the development of novel and efficient antibiofilm solutions.

## Data Availability

No new data were created.
